# Is annual screening by fecal immunochemical test necessary after a recent colonoscopy?

**DOI:** 10.1002/deo2.385

**Published:** 2024-05-20

**Authors:** Yutaka Okagawa, Tetsuya Sumiyoshi, Kota Hanada, Sota Hirokawa, Yusuke Tomita, Masahiro Yoshida, Takeyoshi Minagawa, Kohtaro Morita, Kei Yane, Hideyuki Ihara, Michiaki Hirayama, Hitoshi Kondo

**Affiliations:** ^1^ Department of Gastroenterology Tonan Hospital Hokkaido Japan

**Keywords:** advanced neoplasia, colon adenoma, fecal immunochemical test, screening, total colonoscopy

## Abstract

**Objective:**

The population‐based colorectal cancer screening guidelines in Japan recommend an annual fecal immunochemical test (FIT). However, there is no consensus on the need for annual FIT screening for patients who recently performed a total colonoscopy (TCS). Therefore, we evaluated the repeated TCS results for patients with positive FIT after a recent TCS to assess the necessity of an annual FIT.

**Methods:**

We reviewed patients with positive FIT in opportunistic screening from April 2017 to March 2022. The patients were divided into two groups: those who had undergone TCS within the previous 5 years (previous TCS group) and those who had not (non‐previous TCS group). We compared the detection rates of advanced neoplasia and colorectal cancer between the two groups.

**Results:**

Of 671 patients, 151 had received TCS within 5 years and 520 had not. The detection rates of advanced neoplasia in the previous TCS and non‐previous TCS groups were 4.6% and 12.1%, respectively (*p* < 0.01), and the colorectal cancer detection rates were 0.7% and 1.5%, respectively (no significant difference). The adenoma detection rates were 33.8% in the previous TCS group and 40.0% in the non‐previous TCS group (no significant difference).

**Conclusions:**

Only a few patients were diagnosed with advanced neoplasia among the patients with FIT positive after a recent TCS. For patients with adenomatous lesions on previous TCS, repeated TCS should be performed according to the surveillance program without an annual FIT. The need for an annual FIT for patients without adenomatous lesions on previous TCS should be prospectively assessed in the future.

## INTRODUCTION

Colorectal cancer (CRC) is the second leading cause of cancer‐related death worldwide, and its incidence is increasing.^1^ In Japan, the prevalence and mortality rates of CRC are also high.^2^ Guaiac‐based fecal occult blood screening reportedly is associated with reduced incidences and mortality of CRC.^3^, ^4^, ^5^ Several studies have also shown the effectiveness of fecal immunochemical test (FIT) for CRC.^6^, ^7^ Chiu et al. reported that a single FIT reduced CRC mortality by 62%.^7^ The 2‐day FIT method is used to perform population‐based CRC screening in Japan.^8^ If the FIT is positive on even 1 day, further examination, including total colonoscopy (TCS) or computed tomographic colonography, is warranted. Currently, TCS is the main procedure in real‐world clinical practice in Japan. Several studies have shown that endoscopic removal of adenomatous polyps prevents the development of CRC and reduces CRC‐related death.^9^, ^10^ Although TCS is useful for direct observation of the colorectal mucosa, the risk of post‐colonoscopy CRC remains because TCS can miss some lesions. Reportedly, half of post‐colonoscopy CRCs have been diagnosed after colorectal lesions had been missed.^11^ The Japanese guidelines recommend appropriate TCS intervals depending on the results of the previous TCS.^12^ As mentioned above, the population‐based CRC screening guidelines in Japan recommend an annual FIT.^8^ Therefore, we often experience cases in which FIT results are positive in an opportunistic screening within a few years of the individual having undergone a TCS. Currently, there is insufficient data supporting the benefit of repeated TCS for patients with positive FIT results after a recent TCS, and there is no consensus on the need for annual FIT screening. This study aimed to retrospectively evaluate the results of repeated TCS for patients with positive FIT results after a recent colonoscopy and assess the necessity of an annual FIT for such cases.

## METHODS

### Study design

This was a single‐center, retrospective cohort study. The study protocol was approved by the Institutional Review Board of Tonan Hospital (approved number 1‐15‐1) and was conducted in compliance with the principles of the Declaration of Helsinki. Although written informed consent was not obtained from the patients because of the study design, all participants were informed that they could opt out on the hospital's website.

### Patients

This study included consecutive patients whose FIT was positive for opportunistic screening at the health screening center of Tonan Hospital in Japan from April 2017 to March 2022. The cutoff value of FIT positivity in our hospital is 100 ng/mL. The inclusion criteria were asymptomatic patients aged ≥40 years whose FIT was positive even if only on 1 day. The exclusion criteria were patients with a history of colectomy for CRC, inflammatory bowel diseases (IBDs), unknown history of TCS within 5 years, TCS intervals of <6 months, and who had not received TCS after positive FIT results or had received TCS at another hospital with unknown details. The patients were divided into two groups: patients who had received TCS within the past 5 years (previous TCS group) and those who had not (non‐previous TCS group). In the previous TCS group, for patients who had undergone a total of ≥2 TCS within 5 years, we used the results of the TCS at the first positive FIT results after the initial TCS. The non‐previous TCS group included patients who had undergone TCS >5 years earlier and patients who had not so far.

### Endoscopic procedures

A total of 17 endoscopists used a high‐resolution colonoscope (CF‐H260AZI, CF‐H290I, CF‐HQ290Z, or PCF‐H290ZI; [Olympus Medical Systems], EC‐590ZW/L, and EC‐600ZW/L [Fujifilm]) with carbon dioxide insufflation to perform the colonoscopies. A polyethylene glycol electrolyte solution containing ascorbic acid (MoviPrep; Ajinomoto) and magnesium citrate (Magcorol P; Horii Pharmaceutical) was used to empty and prepare the bowels. Conscious sedation using intravenous administration of diazepam or midazolam and pentazocine was administered during the examination, as required.

### Outcome measures

We analyzed the medical records and endoscopic images of the patients, including the patients’ backgrounds, comorbidities, use of antithrombotic agents, years from previous TCS, reasons for previous TCS, previous TCS results, and detected polyps results. According to a previous report,^13^ the definition of advanced neoplasia (AN) in this study was advanced adenoma (a tubular adenoma ≥10 mm in size, with a villous histology, or high‐grade dysplasia) or invasive cancer. The principal study outcome was the detection rate of AN and invasive cancer in patients who underwent colonoscopy within 5 years. The secondary outcome was the adenoma detection rate (ADR).

### Statistical analysis

EZR (Saitama Medical Center, Jichi Medical University), a graphical user interface for R v.2.13.0 (R Foundation for Statistical Computing) was used to perform all statistical analyses.^14^ Quantitative variables are expressed as the median, whereas categorical variables are presented as total numbers and percentages. Pearson's chi‐square test and the Mann–Whitney U‐test were performed as appropriate. Values of *p* < 0.05 were accepted as indicating statistical significance.

## RESULTS

### Patients’ characteristics

**FIGURE 1:**
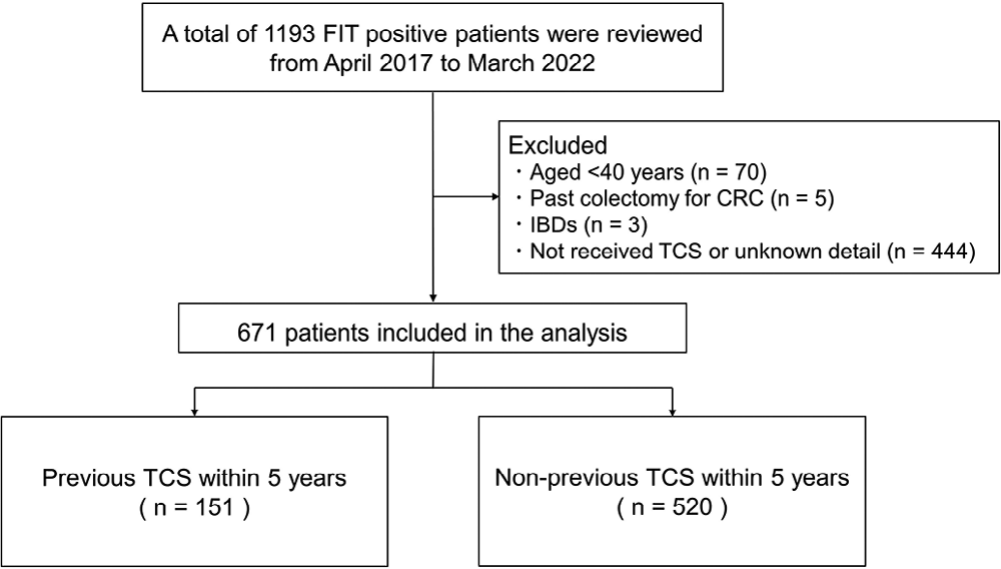
Patient selection flow diagram for the study. Abbreviations: CRC, colorectal cancer; FIT, fecal immunochemical test; IBD, inflammatory bowel disease; TCS, total colonoscopy.

Overall, 24,150 individuals underwent FIT for opportunistic screening at our hospital from April 2017 to March 2022. Of these, 1354 were patients with FIT positive with a positive rate of 5.6% in the screening population. On a patient basis, a total of 1193 patients had ≥1 positive FIT. Of these patients, 522 were excluded for the following reasons: patients aged < 40 (*n* = 70), patients with a past colectomy for CRC (*n* = 5), patients with inflammatory bowel diseases (*n* = 3), and patients who had not received TCS or had received TCS at another hospital with unknown details (*n* = 444). Finally, we analyzed 671 patients (Figure 1). The baseline patient characteristics are presented in Table 1. A total of 151 patients had undergone TCS within 5 years, and 520 had not. In the non‐previous TCS group, 37 patients had undergone TCS >5 years ago. The median age was 59 years in the previous TCS group and 55 years in the non‐previous TCS group, which was a significant difference (*p* < 0.01). The proportion of males was not significantly different between the two groups (*p* = 0.44). There were significant differences in the presence of dyslipidemia (*p* = 0.02) and the medical history of malignancy, excluding CRC (*p* < 0.01). The administration of antithrombotic agents was significantly different between the two groups (*p* = 0.04). In the previous TCS group, 101 patients had received TCS within 3 years and 50 had received TCS within 3–5 years. A positive FIT result was the most common reason for performing previous TCS (62.3%). In the previous TCS, insertion to the cecum was achieved in all patients and all adenomatous lesions were resected. The previous TCS results showed that 104 patients had no adenomatous lesions, 40 had adenomatous lesions excluding AN, and seven had AN.

**TABLE 1:** Patients’ characteristics.

	Previous TCS	Non‐previous TCS	
	*n* = 151	*n* = 520	*p*‐value
Median age, years (range)	59 (41–82)	55 (40–86)	<0.01
Male/female	108/43	350/170	n.s.
Comorbidity			
Hypertension	38	98	n.s.
Dyslipidemia	23	46	0.02
Diabetes mellitus	12	22	n.s.
Past history of malignancy (exc CRC)	19	25	<0.01
Antithrombotic agents	11	18	0.04
Years from previous TCS			
<3 years	101		
3–5 years	50		
Reasons for previous TCS			
Positive FIT	94		
Screening	47		
Surveillance	5		
Others	5		
Results of previous TCS			
No adenomatous lesion	104		
Adenoma (excluding AN)	40		
AN	7		

Abbreviations: AN, advanced neoplasia; CRC, colorectal cancer; FIT, fecal immunochemical test; TCS, total colonoscopy.

### Findings of TCS after a positive FIT result

On TCS after a positive FIT result, the detection rate of AN was 4.6% in the previous TCS group (Table 2), which included one (0.7%) patient with invasive cancer and six patients with advanced adenomas. The patients with invasive cancer had undergone TCS 1 year earlier, and the current TCS showed a laterally spreading tumor (non‐granular type) in the hepatic flexure of the transverse colon, revealing a T1b cancer. The ADR in the previous TCS group was 33.8%. On the other hand, the detection rate of AN was 12.1% in the non‐previous TCS group, which was significantly higher than that in the previous TCS group (*p* < 0.01). The CRC detection rate was 1.5% in the non‐previous TCS group. The ADR of the non‐previous TCS group was 40.0%, which was not significantly different from that of the previous TCS group. In the previous TCS group, 102 adenomas or CRCs were detected. The sigmoid colon was the most common location of detected polyps. The same trend was observed in the non‐previous TCS group (Table 3).

**TABLE 2:** Detection rates of adenoma and advanced neoplasia.

	Previous TCS	Non‐previous TCS	
	*n* = 151	*n* = 520	*p*‐value
Adenoma detection rate	33.8%	40.0%	n.s.
AN detection rate	4.6%	12.1%	<0.01
Invasive cancer detection rate	0.7%	1.5%	n.s.

Abbreviations: AN, advanced neoplasia; TCS, total colonoscopy.

**TABLE 3:** Location of detected lesions.

	Detected adenoma or CRC (%)	
Location	Previous TCS	Non‐previous TCS
Cecum	6 (5.9)	38 (8.4)
Ascending colon	23 (22.5)	72 (15.9)
Transverse colon	21 (20.6)	89 (19.6)
Descending colon	13 (12.7)	59 (13.1)
Sigmoid colon	29 (28.4)	137 (30.3)
Rectum	10 (9.8)	57 (12.6)
Total	102 (100)	452 (100)

Abbreviations: CRC, colorectal cancer; TCS, total colonoscopy.

In the 101 patients who had received TCS within 3 years, the ADR and detection rate of AN were 35.6% and 5.0%, respectively, which were not significantly different from the rates of the patients who had received TCS within 3–5 years (ADR: 30.6%, AN: 4.0%). In the previous TCS group, AN was found in the previous TCS in seven patients. The AN detection rate and ADR in repeat TCS for these patients were 14.3% and 100%, respectively. The rates in patients with adenomas excluding AN in the previous TCS group were 7.5% and 50%, respectively. The rates in patients with no adenomatous lesions in the previous TCS group were 2.8% and 19.2%, respectively (Table 4). The ADR was significantly higher in patients with previous AN than in patients with previous adenoma (excluding AN; *p* < 0.05). Furthermore, the ADR was significantly higher in patients with previous adenoma (excluding AN) than in patients with no previous adenoma (*p* < 0.01). The ADR and AN detection rates were significantly higher in patients with ≥3 adenomatous lesions in the previous TCS than in those without adenomatous lesions. Patients with 1–2 adenomatous lesions in the previous TCS also had higher ADR than those without in the previous TCS (Table S1). Furthermore, when the adenomatous lesions in the previous TCS were divided by size, patients with lesions ≥10 mm had a significantly higher ADR than those with lesions <10 mm or without adenomatous lesions (Table S2).

**TABLE 4:** Comparison of detection rates based on previous total colonoscopy results.

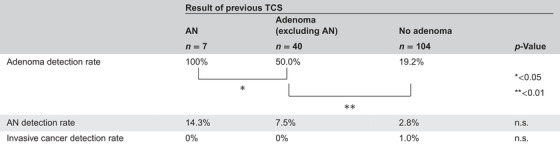

Abbreviations: AN, advanced neoplasia; TCS, total colonoscopy.

## DISCUSSION

The population‐based CRC screening guidelines in Japan recommend the annual 2‐day FIT method^8^; therefore, there are often patients with positive FIT results, so detailed examination is required despite having undergone TCS within a few years. Due to the high efficacy of FIT, the US Multi‐Society Task Force recommends that patients with positive FIT results should be offered a repeated colonoscopy even if a recent colonoscopy was performed, considering the patient background and quality of the previous TCS.^15^ However, this is a weak recommendation based on expert opinion and low‐quality evidence, and there is limited data to inform clinicians on the optimal approach for treating patients with positive FIT results and recent colonoscopies.

In the present study, patients in the previous TCS group were significantly older. This was not surprising, as performing previous TCS was generally thought to be more common in older patients. The significant difference in medical history or administration of antithrombotic agents may have been influenced by differences in age. We analyzed TCS results after positive FIT results in patients who had undergone TCS within 5 years, the detection rate of AN was 4.6%, and the detection rate of CRC was 0.7%. Several studies have reported the association between repeat colonoscopy for patients with positive FIT results and recent colonoscopy.^16^, ^17^, ^18^, ^19^, ^20^ Kim et al. reported the results of TCS for patients aged ≥50 years with positive FIT results.^16^ They found that the detection rates of AN and CRC were 10.9% and 2.1%, respectively, despite having performed TCS within 3 years. Therefore, they suggested repeat colonoscopy for patients with a positive FIT result and a recent TCS, as recommended by the U.S. Multi‐Society Task Force. Another study group also analyzed patients aged ≥50 years with a positive FIT result and a recent TCS and reported that the AN detection rate was 10.3% among those who underwent TCS within 4 years.^17^ They concluded that TCS within 4 years was protective against CRC because CRC was not found in those patients. Kawamura et al. reported the results of patients aged ≥40 years, and the AN and CRC detection rates were 3.9% and 0.3%, respectively, in patients who had undergone TCS within 5 years.^18^ These results are consistent with our own. Furthermore, the researchers suggested that rescreening with a FIT should not be recommended for ≤5 years for average‐risk patients because of the low risk of CRC. On the other hand, Peng et al. reported TCS results for patients with positive FIT results and recent negative TCS findings.^19^ They showed that the CRC incidence per 1000 persons years was 1.34 in patients who had received a subsequent FIT and 2.69 in those who had not, with a corresponding hazard ratio of 0.47. They concluded that a subsequent FIT should be scheduled after negative TCS findings. Whether or not an annual FIT should be administered after TCS remains controversial. Currently, an annual FIT is recommended, and when an FIT result is positive after a TCS, a repeat TCS must be performed. However, if the average detection rates of AN and CRC are as low as our results or those of Kawamura, there would be little benefit in performing an annual FIT after TCS.

In the current study, there was no significant difference in the detection rates of AN between patients who had undergone TCS within 3 years and those who had undergone TCS within 3–5 years. The detected AN might be a missed lesion or a rapidly developing lesion.^20^ Because of the relatively short period of 5 years, the detected ANs will probably be missed lesions rather than rapidly developing lesions. Therefore, we assume that there was no difference in the AN detection rate even when the time periods were separated. Previous reports suggest that TCS should be repeated when there is concern about the quality of the previous TCS or in high‐risk patients, such as older age or family history of CRC.^17^ In our study, the results of a previous TCS affected those of a TCS after a positive FIT result, and patients with previous AN, ≥3 adenomatous lesions, and size ≥10 mm showed higher ADR and AN detection rates in TCS after FIT. Reportedly, the results of a previous TCS can affect the results of surveillance TCS.^21^ Considering these results, an annual FIT is not warranted in patients with adenoma, especially AN, and TCS at intervals in accordance with colonoscopy screening and surveillance guidelines^12^ is preferred. In addition, in patients with no adenoma on the previous TCS, some invasive cancers or ANs were detected on repeated TCS after positive FIT results. Therefore, in the future, it would be necessary to verify the usefulness of FIT in patients with no adenoma detected on the previous TCS.

There were several limitations in this study that should be considered when interpreting the results. First, this was a single‐center retrospective study; therefore, we could not conduct detailed investigations of the patients’ backgrounds, such as family history of CRC. Furthermore, we could not evaluate the quality indicators of TCS, such as bowel preparation quality and ADR of each endoscopist. Second, this was not a population‐based study, so there was some degree of selection bias. Therefore, this study could not be stratified by risk of patient background or previous TCS results. Furthermore, approximately 37% of patients with FIT positive were excluded because they had not received TCS or had received TCS at another hospital and had incomplete information. This study suffered from a severe selection bias. Third, the number of patients in this study was small. Fourth, adenomatous lesions, but not serrated lesions, were examined in this study. Finally, because this was not a prospective study, we were unable to specify whether or not an annual FIT after the recent TCS was necessary.

In conclusion, although some patients were diagnosed with AN or invasive cancer among the patients with FIT‐positive results and who had undergone TCS within 5 years, the detection rate was low, which suggests that an annual FIT after TCS is unnecessary. Repeated TCS should be performed at intervals according to current guidelines without an annual FIT, particularly in patients with adenomatous lesions on previous TCS. In the future, the necessity of an annual FIT for patients with no adenomatous lesion found in a previous TCS should be assessed in light of our findings. Multicenter prospective studies are warranted to address this issue.

## CONFLICT OF INTEREST STATEMENT

None.

## Supporting information


**TABLE S1** Comparison of detection rates based on the number of adenomas in the previous TCS.
Abbreviations: TCS, total colonoscopy; AN, advanced neoplasia
**TABLE S2** Comparison of detection rates based on the size of adenomas in the previous TCS.
Abbreviations: TCS, total colonoscopy; AN, advanced neoplasia
