# Supplementary material for: Is annual screening by fecal immunochemical test necessary after a recent colonoscopy?
Source: DEN Open. 2024 May 20;5(1):e385. doi: 10.1002/deo2.385 (PMC11103454; doi:10.1002/deo2.385)
Supplement: Supplementary file 1 — TABLE S1 Comparison of detection rates based on the number of adenomas in the previous TCS. Abbreviations: TCS, total colonoscopy; AN, advanced neoplasia TABLE S2 Comparison of detection rates based on the size of adenomas in the previous TCS. Abbreviations: TCS, total colonoscopy; AN, advanced neoplasia [file DEO2-5-e385-s001.docx]

Supplemental Table 1. Comparison of detection rates based on the number of adenomas in the previous TCS

|  | | Number of adenomas in previous TCS | | | | | | |
| --- | --- | --- | --- | --- | --- | --- | --- | --- |
|  | | ≥ 3 | | < 2 | No adenoma | | |  |
|  | | n = 13 | | n = 34 | n = 104 | | | p-value |
| Adenoma detection rate | | 76.9% | | 50.0% | 19.2% | | |  |
|  | | * | | ** |  | | | *<0.01 |
|  | |  | |  |  | | | **<0.01 |
| AN detection rate | | 15.4%  *** | | 5.9 | 2.8% | | |  |
|  | |  | |  |  | | | ***0.04 |
| Invasive cancer detection rate | | 0% | | 0% | 1.0% | | | n.s. |
|  |  | |  | | |  |  | |

Abbreviations: TCS; total colonoscopy, AN; advanced neoplasia

Supplemental Table 2. Comparison of detection rates based on the size of adenomas in the previous TCS

|  | | Size of adenomas in previous TCS | | | | | | |
| --- | --- | --- | --- | --- | --- | --- | --- | --- |
|  | | ≥ 10mm | | < 10mm | No adenoma | | |  |
|  | | n = 6 | | n = 41 | n = 104 | | | p-value |
| Adenoma detection rate | | 100% | | 51.2% | 19.2% | | |  |
|  | | * | | ** |  | | | *0.03 |
|  | |  | |  |  | | | **<0.01 |
| AN detection rate | | 16.7% | | 7.3% | 2.8% | | | n.s. |
| Invasive cancer detection rate | | 0% | | 0% | 1.0% | | | n.s. |
|  |  | |  | | |  |  | |

Abbreviations: TCS; total colonoscopy, AN; advanced neoplasia
